# Cornea donation process and tissue quality for transplantation

**DOI:** 10.1371/journal.pone.0249927

**Published:** 2021-04-20

**Authors:** Giovanna Karinny Pereira Cruz, Marcos Antonio Ferreira Júnior, Oleci Pereira Frota, Elen Ferraz Teston, Viviane Euzébia Pereira Santos, Allyne Fortes Vitor, Mayk Penza Cardoso, Fábio Rogério Rodrigues Leocates de Moraes

**Affiliations:** 1 Health Technical School, Federal University of Paraíba, João Pessoa, Paraíba, Brazil; 2 Nursing Department, Health and Development in The Midwest Region, Federal University of Mato Grosso do Sul, Campo Grande, Mato Grosso do Sul, Brazil; 3 Nursing Department, Federal University of Mato Grosso do Sul, Campo Grande, Mato Grosso do Sul, Brazil; 4 Nursing Department, Federal University of Rio Grande do Norte, Natal, Rio Grande do Norte, Brazil; University of Toledo, UNITED STATES

## Abstract

**Introduction:**

The quality of the corneal tissue can be influenced by several factors inherent to the recipient, donor, and to the donation and transplantation process. The donated corneal tissue can be classified by its quality as excellent, good, regular, bad, or unacceptable for transplantation, evaluating it in a slit lamp.

**Objective:**

To analyze the relationship between the clinical and sociodemographic variables of the donors and the donation process and the classification of the quality of the corneal tissue collected for transplantation.

**Methods:**

This is an epidemiologic study, retrospective cohort type, which addressed the process of cornea donation by the Human Eye Tissue Bank in a reference service in Northeast Brazil. The sample consisted of corneas processed by the Human Eye Tissue Bank of Rio Grande do Norte (n = 419). For descriptive and inferential analysis, the study used the *Statistical Package for the Social Sciences* (SPSS) software, version 25.0, and considered a significance level of 0.05. Logistic regression analysis was used for the adjustment of the final model.

**Results:**

It was verified that the epidemiological profile showed a prevalence of individuals with a mean age of 42.54 years old, male (73.99%), and living in the metropolitan region of the state capital (75.66%). When analyzing the relationship between the clinical and sociodemographic variables of the donors, it was identified that those aged 45 years old or less had better quality corneas (excellent and good), while the chronological variables were predictive factors for corneas of regular and bad qualities.

**Conclusion:**

The identification of the factors inherent to the donation process and predictors of corneal tissue quality contribute to minimizing the risk of transplantation and to a better ocular prognosis.

## Introduction

The long-term success of the corneal transplant procedure is highly dependent on the quality of the tissue used. With the current situation showing a great gap between the demand and supply of this type of tissue, obtaining better quality corneas becomes an even greater challenge as the quality of the tissues received by any eye bank depends on several factors [1].

The Human Eye Tissue Banks (HETBs) play an essential role in the search, acquisition, preservation and distribution of corneas for transplantation. Brazil considered corneal donors those with a minimum age of two or more and a maximum of eighty years. This maximum age is due to changes in the corneas resulting from aging (BRASIL, 2009) [2–4].

The increased demand for transplants is accompanied by strict quality control of tissues in the HETBs. This quality control begins with the donor selection process, as well as the use of appropriate techniques for enucleation of the eyeball, preservation of the corneas and evaluation of important parameters, such as the donor serology and the count of endothelial cells present in the tissue. The good quality of the donated cornea and the proper maintenance of the tissue until its use are of fundamental importance for a good final visual prognosis [2–4].

Several studies have pointed out that the results of corneal transplants can be closely related to the quality of the tissues released for transplants, linked to the processes of acquisition, preservation, reevaluation and release of tissues by the HETBs [2, 5–10].

This study aims to analyze the relationship between the clinical and sociodemographic variables of the donors and donation process and the classification of the quality of the corneal tissue acquired for transplantation.

## Material and methods

An epidemiologic observational study with a quantitative approach, of the **retrospective cohort**, longitudinal, descriptive and analytical type that covered the entire cornea donation process by the HETB in the state of Rio Grande do Norte (RN), in the northeastern region of Brazil.

The population consisted of all patients whose corneas were processed by the HETB between 2005 and 2016, over a 12-year period, totaling 3,707 corneas processed.

The sample analyzed was sized by means of sample calculation using a formula for finite populations [11], with a resulting sample of 349 corneas. A percentage of 20% was added referring to the possible losses that could occur, which totaled a final sample of 419 corneas. The simple random sampling method was used to select the final sample analyzed.

The cohort consisted of data of patients whose corneas captured by the HETB of the state of Rio Grande do Norte had the following conditions: corneas registered and classified regardless of the final outcome (excellent, good, regular or poor), provided they were subjected to at least two biomicroscopic evaluations using the slit lamp method, and corneas from donors of any age.

The researchers excluded those whose donor records were not located or were incomplete, inconclusive or illegible, as well as those cases of corneas that, after the first slit lamp examination, were classified as unacceptable for use in transplantation due to follow-up loss for outcome analysis.

The follow-up time for the data collection from the cohort was from the identification of the donor’s time of death until the cornea was released in the eye bank for transplantation.

Data collection was carried out from during the first semester of 2018 after approval of the research protocol by the Research Ethics Committee of the Federal University of Rio Grande do Norte, under Certificate of Presentation of Ethical Appraisal No. 80007117.8.0000.5537 and favorable opinion No. 2,454,077.

The study involved the use of donated tissue from vulnerable populations (children). Brazil considers corneal donors those with a minimum age of two or more and a maximum of eighty years, thus, donors under 18 years old were included in the sample since the exclusion of this group could compromise the analysis and results of the study. We accessed the medical records from January to June 2018. All data were anonymous before accessing them. We collected the data after the written informed consent of those responsible for the medical records.

For data collection, the corneal quality classification was considered as an outcome, made after two biomicroscopic evaluations by ophthalmologists, when the tissues were classified as “excellent”, “good”, “regular” or “bad” for keratoplasty.

For the “corneal quality” variable, the classification given by the eye bank team was considered after evaluating the corneal tissue in a slit lamp at two moments. The first evaluation moment occurred after the enucleation and immersion of the corneal tissue in a preservation medium. The second moment corresponded to the final evaluation of the tissue to release the cornea for transplantation or disposal. The evaluation of the corneal tissue can only be performed by professionals trained by the Pan-American Association of Eye Banks (PAAEB); therefore, all corneas in this study were evaluated by professionals qualified for this purpose.

The classification of the quality of the cornea, attributed by the ophthalmologists of the Eye Bank under study after the evaluations took into account thirteen criteria, namely: senile arch, scars, epithelial defect, epithelial exposure, stromal infiltrate, subepithelial opacity, pterygium, Descemet folds, stromal edema, stromal streak, guttata, specular reflex, and loss of endothelial cells. The ophthalmological criteria used were chosen by the PAAEB and by the Eye Bank Association of America (EBAA) as requirements that make up the evaluation of corneal tissue in the HMETBs [12].

For descriptive analysis of the data, the *Statistical Package for the Social Sciences* (SPSS), version 25.0, was used. To calculate the probability of association between the characteristics analyzed, the bivariate analyses used the Chi-Square test and *Fisher’s* Exact test for the categorical variables and the *Student’s t test* for the quantitative variables, according to each case, as well as measures of magnitude of effect by calculating the Relative Risk (RR). ROC curves were used to identify the cutoff points of the quantitative variables with statistical significance in the prediction of corneal quality. After the bivariate analysis, a final adjustment model was built using logistic regression, using the *Backward Wald Stepwise* method. The significance level considered for all the analyses was 0.05.

## Results

Of the 419 corneas donated, 73.99% of the donors were male, 70.64% were brown-skinned, 45.35% were born in the metropolitan region of the state capital, and 75.66% lived in the Greater Natal area. The mean age of the donors was 42.54 years old (median: 45 years old). The minimum and maximum age of the donors were two and 75 years old, respectively. **Table 1** presents the clinical profile of the 419 cornea donors registered in the HETB.

**Table 1:** Clinical profile of the cornea donors. Brazil, 2020 (n = 419).

Variable	n	%
**Donated cornea**		
Right	212	50.60
Left	207	49.40
**Physiology of death**		
Cardiac arrest	362	86.40
Brain death	57	13.60
**Cause of death**		
Traumatic	235	56.09
Non-traumatic	184	43.91
**Previous ophthalmological surgery**		
Yes	03	0.72
No	416	99.28
**Total**	**419**	**100.00**

After clinical evaluation of the donors, enucleation of the eyeballs was performed. Following enucleation and removal of the corneal scleral button, 82.82% of the tissues were immersed in an Optisol preservation medium, while the rest were immersed in Eusol (17.18%), both hypothermic preservation media (2 to 8°C). Most of the corneas (73.75%) after evaluation in a slit lamp were indicated for optical transplantation. The quality of the corneal tissue evaluated in a slit lamp showed excellent (1.91%), good (51.79%), regular (20.29%), and poor (26.01%) final ratings. Of the total 419 corneas destined for transplants, 72 (17.18%) were later rejected due to their poor quality or to expired date.

The donation process was observed regarding the time intervals between death-enucleation, death-preservation, death-release of the tissue for Corneal Transplant (CT), enucleation-preservation, corneal preservation time, and time elapsed between the first corneal tissue evaluation in slit lamp and the second evaluation, as presented in **Table 2.**

**Table 2:** Chronological variables of the cornea donation process. Brazil, 2020 (n = 419).

Variable	Minimum	Maximum	Median	Mean	SD
Donor’s age (in years old)	2.00	75.00	45.00	42.54	16.22
Death-Enucleation (Minutes)	10.00	1,435.00	228.00	268.43	203.31
Death-Preservation (Minutes)	65.00	1,385.00	349.50	387.82	246.04
Enucleation-Preservation (Minutes)	10.00	942.00	70.00	129.60	167.03
Preservation (Days)	1.00	26.00	9.00	9.19	3.03
Assessment 1 and 2 (Days)	1.00	7.00	1.00	2.07	1.47
Death-Release (Days)	2.00	26.00	9.00	9.51	3.09

*Key*: SD: Standard Deviation.

Regarding the chronological variables of the donation process, the following time interval means were obtained: death-enucleation (4 hours and 28 minutes), death-preservation (6 hours and 28 minutes), and enucleation-preservation (2 hours and 10 minutes). The mean time of immersion of the cornea in the preservation medium was approximately nine days. The time interval between death and release of the cornea from the HETB for transplantation averaged nine and a half days. Regarding the evaluation of the corneas in a slit lamp by ophthalmologists, it was verified that the time between evaluations 1 and 2 was approximately two days.

For the analysis of the statistical probability of association, the “corneal quality” variable was isolated, which corresponds to the classification of the corneal quality after evaluating the tissue in a slit lamp at two moments. The “corneal quality” variable was stratified to form a dichotomous variable, where excellent and good corneas comprised a group and corneas classified as regular and bad constituted the second group of the variable (**Table 3**).

**Table 3:** Corneal quality *versus* demographic and clinical data, Brazil, 2020 (n = 419).

Variables	Classification		Total	Relative Risk [95% CI*]	*p*
	Excellent/Good	Regular/Bad			
	n (%)	n (%)	n		
**Gender**					
Male	169 (54.52)	141 (45.48)	310	0.93 [0.74; 1.17]	0.572 ^(1)^
Female	56 (51.38)	53 (48.62)	109		
**Age group**					
≤ 45 years old	144 (65.75)	75 (34.25)	219	0.58 [0.46; 0.71]	**<0.001**^**(1)**^
> 45 years old	81 (40.50)	119 (59.50)	200		
**Donated cornea**					
Right	120 (56.60)	92 (43.40)	212	0.88 [0.72; 1.08]	0.228 ^(1)^
Left	105 (50.72)	102 (49.28)	207		
**Physiology of death**					
Cardiac arrest	197 (54.42)	165 (45.58)	362	0.90 [0.68; 1.18]	0.456 ^(1)^
Brain death	28 (49.12)	29 (50.88)	57		
**Cause of death**					
Traumatic	146 (62.13)	89 (37.87)	235	0.66 [0.54; 0.82]	**<0.001** ^**(1)**^
Non-traumatic	79 (42.93)	105 (57.07)	184		
**Preservation medium**					
Optisol	187 (53.89)	160 (46.11)	347	0.98 [0.75; 1.28]	0.863 ^(1)^
Eusol	38 (52.78)	34 (47.22)	72		
**Previous surgery**					
Yes	---	03 (100.00)	03	2.18 [1.96; 2.42]	0.098 ^(2)^
No	225 (54.09)	191 (45.91)	416		

Key:

^(1)^Chi-square test.

^(2)^Fisher’s exact test.

*CI: Confidence interval.

Evidence of statistically significant difference was found between the “corneal quality” variable and the donor’s age and cause of death.

The calculation of the measures of magnitude and effect, for the variables whose inferential analysis obtained statistical significance, identified the following relative risks: Donors aged 45 years old or less had a 58% decrease in the risk of having bad or regular corneas when compared to patients over 45 years old. Donors whose deaths were due to traumatic causes had a 66% decreased risk of having bad or regular corneas when compared to patients with non-traumatic deaths.

The chronological variables were evaluated for their relationship with the quality of the corneal tissue, as shown in **Table 4** below.

**Table 4:** Corneal quality *versus* chronological variables. Brazil, 2020 (n = 419).

	Corneal classification	Minimum	Maximum	Median	Mean	SD	CV	*p*
**Death-Enucleation** (in minutes)	**1**	10.00	1,345.00	205.00	244.73	177.93	72.70	**0.010**
	**2**	15.00	1,435.00	265.00	296.07	226.75	76.59	
**Death-Preservation** (in minutes)	**1**	65.00	1,380.00	292.00	344.77	217.20	63.00	**< 0.001**
	**2**	75.00	1,385.00	390.00	438.00	267.83	61.15	
**Enucleation-Preservation** (in minutes)	**1**	10.00	870.00	60.00	106.44	139.84	131.38	**0.002**
	**2**	15.00	942.00	90.00	156.60	190.84	121.87	
**Preservation** (in days)	**1**	3.00	18.00	9.00	9.04	2.58	28.52	0.284
	**2**	1.00	26.00	9.00	9.45	3.72	39.31	
**Death-Release** (in days)	**1**	3.00	19.00	9.00	9.38	2.66	28.40	0.336
	**2**	2.00	26.00	9.50	9.75	3.74	38.40	

*Key*: SD: Standard Deviation. CV: Coefficient of Variation. (1)Excellent/Good. (2)Regular/Bad. SD: Standard Deviation; CV: Coefficient of Variation. *Student’s t test*.

Evidence of statistically significant differences was found between the quality of the cornea and the death-enucleation time, the death-preservation time, and the enucleation-preservation time. The corneas evaluated as excellent or good showed a shorter death-preservation, death-enucleation and enucleation-preservation times.

After the statistical relationship between the chronological variables and the quality of the cornea was identified, the ROC curve was used to verify the respective statistically significant cutoff points to establish the relationship between the time intervals with corneal classification as regular/bad or excellent/good. The analysis verified that the time intervals between death and preservation greater than 385 minutes (6 hours and 25 minutes), death-enucleation greater than 205 minutes (3 hours and 25 minutes), and enucleation-preservation greater than 85 minutes (1 hour and 25 minutes) were related to corneal tissues of regular and poor quality.

After the bivariate analysis, multivariate analysis was started by performing multiple logistic regression. The inclusion criterion of the explanatory variables with a p-value<0.20 used in bivariate analysis previously carried out was adopted for the application of multiple logistic regression. Thus, the following variables were selected to choose the model: time interval between death and enucleation, time interval between death and preservation, time interval between enucleation and preservation, age group, cause of death, and previous surgery. The dependent variable considered was “corneal quality”, coded as “excellent/good (0) and regular/poor (1)”.

Thus, logistic regression made it possible to identify the odds ratios of the variables that made up the final model with the quality of the graft. The final logistic regression model (*Backward Wald Stepwise* method), for a significance value of 5%, contemplated the “age group” variable. It was verified that the chance of donors aged less than or equal to 45 years old of having a cornea classified as regular or bad decreased by 74% when compared to donors aged over 45 years old.

## Discussion

Due to the limited number of corneas donated and available in many regions of the world, the corneal donation-transplantation process evolves rapidly in order to optimize clinical results and to protect the vision of the tissue receptors [13].

The quality control adopted by the HETBs starts with the use of appropriate corneal acquisition and preservation techniques, with the evaluation of medical contraindications, the performance of donor serology tests and the evaluation of the quality of the donated tissue through biomicroscopic analysis, performed in a slit lamp [14].

The quality of corneal graft is one of the main criteria for defining the success of corneal transplant [15]. After statistical analysis, it was observed that the analysis of this quality presented as predictive factors the cause of death, the age of the donor, and the time intervals between death and enucleation, death and preservation, and enucleation and preservation.

It was identified that, in cases of death due to traumatic causes, the risk of having a bad or regular cornea was reduced by 66% when compared to those with non-traumatic deaths. The literature does not corroborate these results. A cohort study conducted in Brazil with 3,388 cornea donors showed that corneas from donors with violent deaths did not present any statistically significant interference with respect to tissue quality [16]. The association established between the cause death and the quality of the corneal tissue may have been identified due to the donor’s age, since the majority of victims of traumatic deaths are young adults, justified by the mean age of the cornea donors and male gender highlighted in this study.

Young adults are the main victims of deaths due to external causes in Brazil. Traffic accidents, homicides and suicides together account for nearly two thirds of the deaths due to external causes in Brazil. Rates are considerably higher in the population of young adults, mainly males [17].

Regarding the age of the donor, it was observed that donors aged 45 years old or less had a 58% decrease in the risk of having a bad or regular cornea compared to patients over the age of 45.

A study carried out in the USA with the aim of evaluating the effect of donor age on graft survival and loss of endothelial cells in penetrating keratoplasty observed that, after 5 years, there was no difference in graft survival (86%) among the participants who received corneas of donors aged 12–65 and 66–75 [18]. On the other hand, studies in the United Kingdom, India and Brazil identified that the donor’s advanced age was associated with the quality of the cornea [1, 10, 19, 20]. It was possible to identify that, for each one-year increase in the donor’s age, there is 1% fewer chances of the cornea being released for CT, which shows the relationship established between the donor’s age and the quality of the graft [20].

Thus, according to the results of this study, it is suggested that the donor’s advanced age corresponds to a predictive factor for the decrease in the quality of corneal tissue. This premise is justified by the endothelial dysfunction that occurs over the years. The greater the age, the smaller the number of corneal endothelial cells, which leads to corneal tissue endothelial dysfunction and to a consequent corneal parenchyma impairment [2, 21, 22].

The quality of the corneas is linked to intrinsic factors of the donor and to the acquisition and processing of these tissues before their release for transplantation or disposal. The donor’s age can be related to the quality of the corneal tissue due to changes in the corneal endothelium in the aging process. However, due to the changes in the age pyramid, with the increase in life expectancy, the contraindication of donors with advanced ages would not be effective, since the proportion of elderly donors is higher when compared to younger age groups. Therefore, when assessing the quality of the corneal tissue, the density of the endothelial cells should be considered as an indicator of tissue quality and surgical indication [10, 15, 19].

Currently, there is a discrepancy in the acceptable limits regarding the time between death and preservation of the cornea. Some studies claim that there are no upper time limits, that is, as long as certain quality criteria are met, the corneas can be used for grafting. In the eye tissue banks in the world, tropical countries with hot and humid climates adopt six hours as a cutoff point for the preservation of non-refrigerated bodies. In developing countries, it has been verified that, in most of the tissues, enucleation of the eyeball occurs within six hours after death, as it happens in Brazil [23, 24].

The chronological factors of the donation process vary according to the period studied, the places where the donations occur, the preservation media used, and the environmental and cultural factors, as verified in published studies [14, 19, 20, 23, 25]. Therefore, it is essential to determine to which extent the chronological variables can affect the quality of the graft, in order to obtain the proper management of these determinants during the donation process and, consequently, to guarantee better quality corneal tissues.

In this study, an association was verified between the quality of the corneal tissue and the time interval between death and enucleation, death and preservation, and enucleation and preservation. The statistical analysis of the data identified that the time intervals between death and enucleation greater than 205 minutes (3 hours and 25 minutes), between death and preservation greater than 385 minutes (6 hours and 25 minutes) and enucleation-preservation greater than 85 minutes (1 hour and 25 minutes) showed a statistically significant relationship with corneal tissue of regular and bad qualities.

The process of extracting the tissue should be agile without compromising the safety and maintenance of the technique for preserving the tissue. A study carried out in India identified that corneas donated with a time between death and preservation of 6 to 10 hours can be used in transplants for optical purposes, as long as they meet the acceptance criteria of the tissue for optical use. In Brazil, another study pointed out that the time interval between death and preservation less than or equal to 5 hours and 45 minutes was associated with better quality of the corneal tissue [10, 24].

A number of research studies have shown that shorter intervals during the donation process have brought positive results to the donated tissue. The early extraction of corneal tissue allowed to prevent *post-mortem* infection and to promote conservation in adequate conditions of preservation and temperature, fundamental conducts for maintaining the healthy characteristics of the tissue [14, 20, 25, 26].

It is suspected that the degradation of the ocular content caused by the donor’s death may alter the aqueous humor in order to accelerate the death of the endothelial cells. This suggests that, the faster the eye is removed from the corpse and the cornea from the eye, the lower the chance of endothelial poisoning. To minimize tissue damage, it is recommended to keep the eyelids closed from asystole to extraction, as well as to cool the body as soon as possible [14, 20, 25–27].

Over the past decade, changes in the approach to tissue extraction using the conventional globe-wide enucleation technique for *in situ* excision of the corneas (corneal-scleral button) have resulted in best quality corneas. With regard to the cornea donation process, this change helped to shorten the corneal processing time between death and preservation and ensured better quality tissues [14].

The current pandemic context of SARS-CoV-2 worsened the high demand for people on the waiting list for corneal transplantation. This scenario emphasized the discussion and search for alternative and safe methods of using corneas without wasting low-quality tissues, such as dehydration techniques of the corneal stroma for a long time without compromising the tissue for transplants of the anterior portion of the cornea or tectonics; the use of peripheral endothelial cells with proliferative potential in cell culture to ensure a substantial increase in the donor endothelial cell pool for regenerative treatments; and the adoption of lamellar techniques for dividing the donor cornea into multiple grafts for multiple recipients [28–31].

Corneal transplantation has evolved considerably but an adequate supply of viable tissues to meet the growing demand is still a priority for the specialty. We believe that this is the time to rethink allocation policies to facilitate the division of the donor cornea into multiple grafts for multiple recipients. We need to act on time, as we expect an increase in the next 12 to 24 months since it is likely that we will have to live with SARS-CoV-2 for at least that time [31].

The analysis of the clinical and sociodemographic factors of the donation process associated with the quality of the corneal tissue showed the importance of implementing HETB quality control programs, in order to promote the selection of corneal tissues with good quality and guarantee a donation process with mechanisms for identifying donors, extracting, preserving and distributing corneal tissue guided by good practices that seek to minimize the risk of compromising tissue quality.

The quality of the corneal tissue is a fundamental factor for the success of the transplant. In order to guarantee good quality tissues, it is important that the time limits between death and enucleation, death and preservation, and enucleation and preservation are established by the HETB regulatory institutions, in order to minimize the risks to which the tissues are exposed due to chronological factors related to the donation process.

## Abbreviations page

PAAEB: Pan-American Association of Eye Banks
HETBs: Human Eye Tissue Banks
USA: United States of America
EBAA: Eye Bank Association of America
RN: Rio Grande do Norte
RR: Relative Risk
SPSS: Statistical Package for the Social Sciences Software
CT: Corneal Transplant
